# Multiobjective Binary Differential Approach with Parameter Tuning for Discovering Business Process Models: MoD-ProM

**DOI:** 10.1155/2024/9545184

**Published:** 2024-08-27

**Authors:** A. Sonia Deshmukh, B. Shikha Gupta, C. Naveen Kumar

**Affiliations:** ^1^ Department of Computer Science and Information Technology KIET Group of Institutions, Ghaziabad, Uttar Pradesh, India; ^2^ Department of Computer Science S.S. College of Business Studies University of Delhi, New Delhi, Delhi, India; ^3^ Department of Computer Science University of Delhi, New Delhi, Delhi, India

## Abstract

Process discovery approaches analyze the business data to automatically uncover structured information, known as a process model. The quality of a process model is measured using quality dimensions, completeness (replay fitness), preciseness, simplicity, and generalization. Traditional process discovery algorithms usually output a single process model. A single model may not accurately capture the observed behavior and overfit the training data. We have formed the process discovery problem in a multiobjective framework that yields several candidate solutions for the end user who can pick a suitable model based on the local environmental constraints (possibly varying). We consider the Binary Differential Evolution approach in a multiobjective framework for the task of process discovery. The proposed method employs dichotomous crossover/mutation operators. The parameters are tuned using grey relational analysis combined with the Taguchi approach. We have compared the proposed approach with the well-known single-objective algorithms and state-of-the-art multiobjective evolutionary algorithm—Nondominated Sorting Genetic Algorithm (NSGA-II). Additional comparison via computing a weighted average of the quality dimensions is also undertaken. Results show that the proposed algorithm is computationally efficient and produces diversified candidate solutions that score high on the fitness functions. It is shown that the process models generated by the proposed approach are superior to or at least as good as those generated by the state-of-the-art algorithms.

## 1. Introduction

Processes are ubiquitous in any organization. An efficient organization is built on processes that run in a symphony to achieve growth and customer/employee satisfaction. In the present digital era, organizations maintain the process execution information in the form of transaction logs that are amenable to analyses. However, amidst routine activities, an organization may not analyze the effectiveness of the processes being followed. Process mining aims to extract nontrivial knowledge and exciting insights from data recorded by the information systems, stored in the form of an event log. In the past decade, process mining adoption has expanded considerably, evidenced by numerous industry and academic use cases, especially in auditing and healthcare, with the field maturing through enhanced tools and techniques [1, 2]. The prominent process mining challenges include process discovery, conformance checking, and enhancement. Process discovery algorithms build a process model from the given event log [3–5]. Conformance checking verifies the goodness of the discovered process models. Enhancement techniques extend or improve existing processes by identifying and removing bottlenecks, finding deviations, recommending adjustments, and repairing processes using the information in an event log [3, 4]. The present work is focused on the challenge of the process discovery.

Process discovery concerns itself with extracting information on existing processes to recognize the bottlenecks, deviations, and inefficiencies in the day-to-day process workflows, providing concrete steps towards business process improvement. The last decade has seen several process discovery techniques that optimize one or more quality metrics, namely, completeness (also known as replay fitness [3]), preciseness, simplicity, and generalization or their weighted function. Typically, process discovery algorithms output a single model. However, a single process model may not always describe the recorded behavior of the log effectively and may be a consequence of overfitting the training data.

In this paper, we present multiobjective differential approach in process mining (MoD-ProM), a process discovery algorithm that generates several competing process models, representing different trade-offs in the quality dimensions. The present work formulates process discovery as a multicriterion problem. The proposed approach applies the differential evolution algorithm and optimizes Completeness and Generalization quality metrics to output several candidate process models. Subsequently, the solutions may either be evaluated by a domain expert to best suit the situation at hand or be chosen by the user based on his/her preference.

The contributions of this proposal are

- A novel application of differential evolution approach for discovering a Pareto-front of the process models.
- We adapted a binary version of the multiobjective differential evolution algorithm and used dichotomous operators [6].
- The proposed algorithm (MoD-ProM) is evaluated on ten synthetic and four real-life event logs, and results are compared with the state-of-the-art algorithms.
- The parameters are tuned using grey relational analysis combined with the Taguchi approach [7, 8].
- The computation of fitness functions (completeness and generalization) has been reformulated in terms of the causality relation matrix.

The results reveal that the proposed approach (MoD-ProM) outperforms the compared algorithms regarding the quality of the process model. Compared to Nondominated Sorting Genetic Algorithm II (NSGA-II) [9], the proposed algorithm exhibits a lower computational cost. The competing solutions (Pareto set) generated by the proposed approach are better than the nondominated solutions generated by NSGA-II.


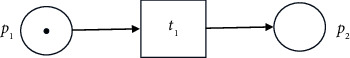


The remainder of this paper is organized as follows: Section 2 outlines the basic concepts related to process discovery and the related work.  Section 3 describes the solution strategy, and Section 4 presents the results of the experiments. Finally, Section 5 gives the conclusion of the paper.

## 2. Background and Related Work

### 2.1. Process Discovery

Process discovery is an evolving domain that leverages event logs to analyze business processes and present factual insights. An event log is the starting point for process discovery algorithms and represents a business process using the case notation to correlate events. A “case” in this notation refers to an instance of a process and is also known as a trace. Each case is assigned a unique ID, called the Case ID. An instance of a process may involve multiple activities or tasks over many days. An occurrence of a task in the context of a particular process instance (case), along with its timestamp, is called an event.  Table 1 gives an example of an event log. In this example, 101, 102, and 103 represent the Case ID of three process instances, and *T*_1_, *T*_2_,…, and *T*_7_ represent the various tasks carried out in the system.

#### 2.1.1. Visualisation of a Process Model

A process model can be discovered from the given event log and may be visualized in various forms such as Business Process Modelling Notation (*BPMN* models), Petri nets, and Data Flow Graphs (*DFGs*). In this paper, the discovered process model is graphically represented as a *Petri net*, a popular method for representation. A Petri net is a bipartite graph, composed of nodes, tokens, and directed arcs. A node could be a place (denoted by a circle) or a transition (denoted by a square). The places and the transitions are joined by directed arcs. For example, in the following figure, *p*_1_ and *p*_2_ are places and *t*_1_ is a transition.

A transition is also called a task. The token is the information that needs to be processed. Each place can hold zero or more tokens. In the above figure, the place *p*_1_ holds a single token. The directed arcs can transfer one token. Transitions cannot store tokens. Arcs connect (input) places to transitions and transitions to (output) places. The state of a Petri net is given by its assignment of tokens to places.

A transition is said to be enabled if each input place holds at least one token. In the following figure, *t*_1_ transition is enabled.


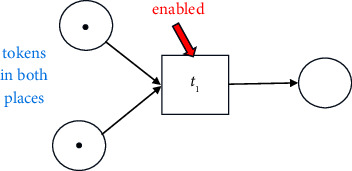


An enabled transition may fire at any time. When fired, the tokens in the input places are moved to the output places of the transition. Firing of a transition results in a new state of the Petri net. The following figure shows the change in the above Petri net after transition *t*_1_ fires.


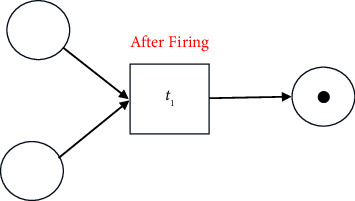


A transition cannot be enabled if a token is absent (missing token) at any input place. For example, in the following figure, transition *t*_1_ cannot be enabled.


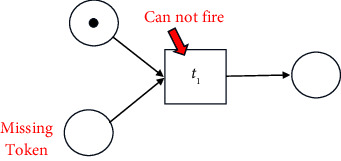


**Figure 1:**
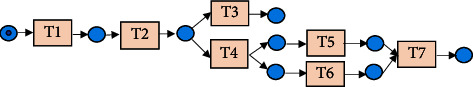
Petri net for the example event log of Table 1.

Figure 1 depicts a Petri net that conforms to the example event log in Table 1.

#### 2.1.2. Well-Known Algorithms for Process Model Discovery

State-of-the-art process discovery techniques include *α* [10], *α*^+^ [11], multiphase miner [12, 13], heuristics miner [14], genetic process mining (GPM) [15], *α*^++^ [16], *α*^#^ [17], *α*^∗^ [18], fuzzy miner [19], and Inductive Logic Programming (ILP) [20] algorithms [21]. Other algorithms in the domain of process discovery include evolutionary tree miner (ETM) [22], inductive miner [23], and multiparadigm miner [24]. Reference [25] proposed a hybrid process mining approach that integrates the GPM, particle swarm optimization (PSO), and discrete differential evolution (DE) techniques to extract process models from event logs. Reference [26] proposed the Fodina algorithm, an extension of the heuristic miner algorithm [26].

Reference [27] proposed an extension of the ETM algorithm [22] that discovers a collection of mutually nondominating process trees using NSGA-II [28]. This algorithm optimizes replay fitness, precision, generalization, simplicity, and the number of low-level edits.

#### 2.1.3. Motivation for the Proposed Algorithm

Usually, state-of-the-art process discovery algorithms output a single process model that may overfit the training data. To capture the observed behavior more accurately, we propose a multiobjective algorithm for process discovery. The proposed approach yields several candidate solutions. Subsequently, the solutions may either be evaluated by a domain expert to best suit the situation at hand or be chosen by the user based on the local environmental constraints (possibly varying).

The proposed algorithm formulates the problem of process model discovery in a multiobjective framework using the differential evolution approach [29]. Differential evolution (DE) is a versatile and stable evolutionary algorithm. It evolves individuals through the perturbation of population members with scaled differences of distinct population members. DE algorithm has consistent robust performance and is suitable for solving various numerical optimization problems [30].

### 2.2. Multiobjective Binary Differential Evolution

The proposed algorithm employs a binary version of the differential evolution approach to suit the process mining domain. While DE was initially designed to operate in continuous space, the authors in [6] proposed a binary DE (BDE) algorithm based on dichotomous mutation and crossover operators. The authors in [6] verified that compared to other notable BDE variants, the dichotomous BDE improves the diversity of the population and enhances the exploration ability in the binary search space. Also, it has been shown that as compared to other BDE variants, the dichotomous algorithm does not involve any additional computation cost and is faster than other variants of BDE [6].

The past decade has seen the application of the DE approach to problems where the optimization of multiple objectives is required. Reference [31] first proposed a DE-based approach for multiobjective real-coded optimization problems. According to [32], in the case of binary-coded optimization problems, multiobjective BDE algorithms explore the decision space more efficiently than other multiobjective evolutionary algorithms. Subsequently, multiobjective BDE algorithms were also proposed [33–37].

## 3. Materials and Methods

A process discovery algorithm is a function that maps an event log onto a process model that best represents the behavior seen in the event log. In the present work, a process model is represented by a causality relation matrix *C* = (*c*_*t*_1_,*t*_2__), where *t*_1_, *t*_2_ ∈ [1, *n*] represent the tasks, *c*_*t*_1_,*t*_2__ ∈ {0, 1}, and *n* is the number of tasks in the given event log. That is, an individual in the population is binary-coded. We, therefore, adapted a binary version of the multiobjective differential evolution algorithm using dichotomous operators [6]. The steps for the proposed multiobjective differential approach for process mining (MoD-ProM) are outlined in Algorithm 9. These steps are explained in the following subsections.

### 3.1. Initialization

The given event log *E* with *n* tasks is first consolidated into a dependency measure matrix *D* indicating the degree of dependencies between tasks [15]. Considering the example in Figure 1, where *T*_1_, *T*_2_,…, and *T*_7_ represent the tasks. A dependency exists between activities *T*_1_ and *T*_2_ if, in a trace, either *T*_1_ directly precedes *T*_2_ or vice versa. This is indicated by the presence of either the strings *T*_1_ *T*_2_ or *T*_2_ *T*_1_ in a process instance (trace) of the event log. The strength of dependency is proportional to the frequency of occurrence of these strings. In the example log (Figure 1), *T*_1_ directly precedes *T*_2_ whereas the string *T*_2_ *T*_1_ does not occur at all. That is, in the given system, task *T*_1_ is more likely to be the cause of task *T*_2_ than vice versa. Dependency measure is computed by counting the length-one-loops (for example, *T*_1_ *T*_2_), self-loops (for example, *T*_1_ *T*_1_), length-two-loops (for example, *T*_1_ *T*_2_ *T*_1_), and parallel tasks (for example, *T*_1_ *T*_2_ and *T*_2_ *T*_1_ occur an equal number of times). In the example log (Figure 1), *T*_5_ and *T*_6_ are parallel tasks and *T*_1_ *T*_2_ is a length-one-loop [38].

As proposed by [39], the present work represents a process model as a causality relation matrix. To represent a process model, [39] have favored a causality relation matrix over the more popular Petri net since representing the population individual as a causality relation matrix makes it easier to initialize the population and define the genetic operators. While a causality relation matrix can be directly derived from the information in the event log, in Petri nets, there are places whose existence cannot be derived directly from the event log [39]. The mapping between a Petri net and a causality relation matrix is detailed in [39]. We have graphically depicted both representations for an example event log in the section titled “Process Model Representation.”

### 3.2. Objective Functions and Fitness Evaluation

In the proposed algorithm, we use a novel combination of completeness and generalization as objective functions. Completeness is an important quality dimension because a discovered process model is expected to describe the behavior stored in the log. Completeness is the process of computation of all the parsed tasks while replaying the traces of the log in the model. The missing tokens in a trace and the extra ones left behind during parsing (unconsumed tokens) contribute to the penalty value. Generalization shows whether the process model accurately represents the system as it is and is not “overfitting” to the behavior observed in the event log [40]. Completeness [15] and generalization [41] are computed as in Algorithms 1 and 2, respectively. The algorithms make use of the following function for a given event log E:

(1) $$\begin{array}{c} \text{follows}\left(t_{1},t_{2},E\right)=\left\{\begin{array}{cc} 1, & \text{if }t_{1}\;t_{2}\text{ is length}-\text{one}-\text{loop in }E,\\ 0, & \text{otherwise}, \end{array}\right. \end{array}$$

(2) $$\begin{array}{c} \text{follow }s_{k}\left(t_{1},t_{2},E\right)=\left\{\begin{array}{cc} 1, & \text{if }t_{2}\text{ is the }k^{th}\text{ task after }t_{1}\text{ in }E,\\ 0, & \text{otherwise}. \end{array}\right. \end{array}$$

The present proposal performs an additional analysis of the discovered process models by evaluating their preciseness and simplicity values. The preciseness value of a model is relative to an event log and quantifies the behavior existing in the model but not observed in the event log [42]. A process model with a high precision value is not expected to show behavior not observed in the event log [43]. Completeness and preciseness only consider the relationship between the event log and the process model. However, just a portion of all potential behavior that the system permits is recorded in the event log. Simplicity, instead of telling about the behavior observed in the event log, shows the internal structure of the discovered model. Preciseness [22] and simplicity [44] values are computed as in Algorithms 3 and 4, respectively.

### 3.3. Constraints and Decision Variables

For a given event log *E*, dependency measure matrix *D*=(*D*(*t*_1_, *t*_2_)) is used to generate causality relation matrices *C*^*i*^ = (*c*_*t*_1_,*t*_2__^*i*^) where ∈[1, *N*] , *t*_1_,  *t*_2_ ∈ [1, *n*], and N is the population size (Algorithm 5). The dependency measure matrix and the causality matrix correspondingly represent the constraints and the decision variables for the problem. Each causality relation matrix represents an individual of the initial population and is computed as [15]

(3) $$\begin{array}{c} c_{t_{1},t_{2}}^{i}=\left\{\begin{array}{cc} 1, & \text{if }r<D\left(t_{1},t_{2}\right),\\ 0, & \text{otherwise}, \end{array}\right. \end{array}$$

*r* ∈ [0, 1) is a random number.

### 3.4. Mutation

For a population member *C*^*i*^ = (*c*_*t*_1_,*t*_2__^*i*^), *i* ∈ [1, *N*] , *t*_1_,  *t*_2_ ∈ [1, *n*], two other causal matrices *C*^*r*_1_^, *C*^*r*_2_^, *r*_1_ ≠ *r*_2_ ≠ *i*, *r*_1_, *r*_2_ ∈ [1, *N*] are chosen randomly from the current population. A mutant individual *V*^*i*^ = (*v*_*t*_1_,*t*_2__^*i*^) is then created using the following dichotomous mutation scheme [6].

(4) $$\begin{array}{c} v_{t_{1},t_{2}}^{i}=\left(\left(c_{t_{1},t_{2}}^{r_{1}}⊕c_{t_{1},t_{2}}^{r_{2}}\right)\wedge \text{rand}\right)\vee \left(\neg \left(c_{t_{1},t_{2}}^{r_{1}}⊕c_{t_{1},t_{2}}^{r_{2}}\right)\wedge c_{t_{1},t_{2}}^{r_{1}}\right) \end{array}$$

where rand ∈{0, 1}, ∧ denotes the AND operator, ∨ denotes the OR operator, ¬ denotes the NOT operator, and ⊕ denotes the XOR operator. (4) can also be expressed as

(5) $$\begin{array}{c} v_{t1,t2}^{i}=\left\{\begin{array}{cc} r\text{and}, & \text{if }c_{t_{1},t_{2}}^{r_{1}}⊕c_{t_{1}{,}_{t}2}^{r_{2}}=1,\\ c_{t_{1},t_{2}}^{r_{1}}, & \text{if }c_{t_{1},t_{2}}^{r_{1}}⊕c_{t_{1},t_{2}}^{r_{2}}=0. \end{array}\right. \end{array}$$

That is, if *c*_*t*_1_,*t*_2__^*r*_1_^ and *c*_*t*_1_,*t*_2__^*r*_2_^ are distinct, then the corresponding bit of the mutant individual *v*_*t*_1_,*t*_2__^*i*^ is randomly chosen as “0” or “1”; otherwise, *v*_*t*_1_,*t*_2__^*i*^ is set as *c*_*t*_1_,*t*_2__^*r*_1_^.

### 3.5. Crossover

The dichotomous crossover operator [6] starts from the mutant individual *V*^*i*^ = *v*_*t*_1_,*t*_2__^*i*^, obtained after application of the dichotomous mutation operator. In this step, the original individual *C*^*i*^ = (*c*_*t*_1_,*t*_2__^*i*^) and the mutated individual *V*^*i*^ are used to generate a candidate individual *U*^*i*^ = *u*_*t*_1_,*t*_2__^*i*^ using the following equation:

(6) $$\begin{array}{c} u_{t_{1},t_{2}}^{i}=\left\{\begin{array}{c} v_{t_{1},t_{2}}^{i},\text{ if }r{\text{and}}_{t_{1},t_{2}}<CR_{t_{1},t_{2}},\\ c_{t_{1},t_{2}}^{i},\text{ otherwise}, \end{array}\right. \end{array}$$

where rand_*t*_1_,*t*_2__∈[0, 1],

(7) $$\begin{array}{c} CR_{t_{1},t_{2}}=\left\{\begin{array}{c} CR_{1},\text{ if }\left(c_{t_{1},t_{2}}^{r_{1}}⊕c_{t_{1},t_{2}}^{r_{2}}\right)=0,\\ CR_{2},\text{ if }\left(c_{t_{1},t_{2}}^{r_{1}}⊕c_{t_{1},t_{2}}^{r_{2}}\right)=1. \end{array}\right. \end{array}$$

This operation uses two crossover probabilities *CR*_1_ and *CR*_2_ based on dichotomous psychological thinking or “black and white” thinking, with a proclivity for only seeing extremes. After mutation, to generate a candidate individual, if the bits in the randomly chosen individuals from the original population are the same (distinct), then crossover probability *CR*_1_(*CR*_2_) is used. This approach induces diversity in the population and enhances the exploration ability of the proposed approach [6].

### 3.6. Selection

In this section, we outline the selection procedure (Algorithm 6) used to determine the individuals to be preserved from the current population Pop = {*C*^1^, *C*^2^,…, *C*^*N*^}, and the candidate population 1 = {*U*^1^, *U*^2^,…, *U*^*N*^} generated after the crossover operation. The process involves identifying the nondominated individuals.

The *i*^*th*^ individual from the current population (parent) (*C*^*i*^) is said to dominate (≺) the corresponding *i*^*th*^ individual in the candidate population (child) (*U*^*i*^) if the parent is superior for both the objectives of completeness and generalization, that is,

(8) $$\begin{array}{c} C^{i}≺U^{i}=\left\{\begin{array}{c} 1\;,\text{ if }f_{c}\left(U^{i}\right)\geq f_{c}\left(C^{i}\right)\;\&\&\;f_{g}\left(U^{i}\right)\geq f_{g}\left(C^{i}\right),\\ 0,\text{ otherwise}, \end{array}\right. \end{array}$$

where *f*_*c*_ and *f*_*g*_ denote the completeness and generalization values, respectively.

If the parent (child) dominates the child (parent), then the parent (child) is preserved, while the child (parent) is discarded. When neither parent nor child is superior to each other, both the parent and the child are retained.

After eliminating dominated individuals, the number of remaining nondominated individuals will be between N and 2 ∗ N. Since the population size to be carried for the next generation is N, a truncation procedure based on nondominated sorting (Algorithm 7) and crowding distance (Algorithm 8) is applied [31].

Nondominated sorting algorithm (Algorithm 7) involves finding rank 1 individuals of the population that are not dominated by any other individual. Rank 2 is assigned to those individuals of the population that are dominated by rank 1 individuals and so on.

If the number of nondominated solutions is greater than the population size N, Euclidean distance is used to truncate individuals from the most crowded region (Algorithm 8). If the rank 1 individuals are less than N, then rank 2 individuals are added and so on.

## 4. Results and Discussion

### 4.1. Experimentation

The proposed algorithm is tested on both synthetic and real-world datasets (Table 2). Over the last decade, BPI challenge event logs have become important real-world benchmarks in the data-driven research area of process mining. The proposed algorithm is tested for three BPI event logs, namely, BPI 2012 [45], BPI 2013 [46], and BPI 2018 [47], varying in the number of tasks, number of traces, and their domain. BPI 2012 is one of the most studied datasets in process mining. This dataset contains 13,087 traces and 23 tasks and is derived from a structured real-life loan application procedure released to the community by a Dutch financial institute. The BPI 2013 dataset is from the IT incident management system of Volvo Belgium with 7554 traces and 13 tasks. BPI 2018 covers the handling of applications for EU direct payments for German farmers from the European Agricultural Guarantee Fund. BPI 2018-reference dataset contains 43802 traces and 6 tasks. The proposed algorithm is also tested on a real-life medical event log containing events of sepsis cases from a hospital with 1000 traces and 16 tasks [48]. The proposed algorithm is also run for synthetic logs (ETM, g2-g10 [15, 38, 44]).

The proposed approach is compared with state-of-the-art algorithms, *α*^++^ [16], Heuristic Miner [14], Genetic Miner [15], ILP [20], and Inductive Miner [23] algorithms. For the compared algorithms, the completeness, preciseness, and simplicity values for the synthetic datasets are taken as reported by [44]. However, the authors in [44] do not report the value of generalization for these datasets. For the models generated using the Prom tool, *α*^++^, Heuristic Miner, Genetic Miner, and ILP algorithms, the generalization value is computed using the Cobefra tool [44, 49]. We have also compared the proposed strategy with the NSGA-II algorithm for process discovery.

In the proposed multiobjective differential approach for process mining (MoD-ProM), the population size is set to 100 and the value of control parameters *CR*_1_ and *CR*_2_ is tuned using grey relational analysis combined with the Taguchi approach (Section).

The algorithm is run for a maximum of 100 iterations as the proposed algorithm converges before 100 iterations for most datasets. The total number of runs is fixed at 30.

### 4.2. Parameter Tuning

**Figure 2:**
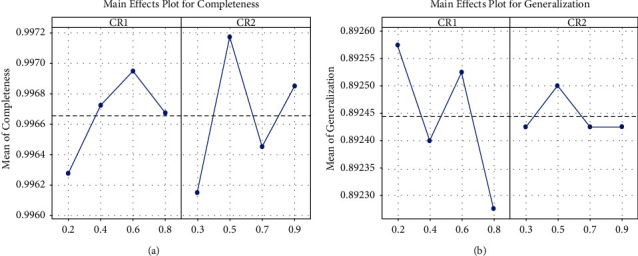
Main effects plot of completeness and generalization.

To find values of the crossover probabilities, *CR*_1_ and *CR*_2_ are suitable for the domain of process discovery and the grey relational analysis combined with the Taguchi approach is used [7, 8]. The Taguchi method efficiently determines optimal settings of numerous process variables with a minimal set of experiments. The Taguchi method suggests replication of the experiment to achieve improved accuracy of the results. Taguchi L16 orthogonal array (OA) design containing 16 experimental runs is used. The results for completeness and generalization are shown in Table 3 and Figure 2. Dr. Taguchi's Signal-to-Noise ratios (S/N), which are log functions of the desired output, serve as objective functions for optimization [50]. The optimization of numerous performance variables requires a comprehensive assessment of the S/N ratio. The grey relational analysis is used in the study to solve this issue [8].

In the grey relational analysis combined with the Taguchi approach, the experimental data are normalized using equation (9) to avoid different units and to reduce the variability as presented in Table 4.

(9) $$\begin{array}{c} x_{i}^{*}\left(k\right)=\frac{x_{i}\left(k\right)-\;\min\left(x_{i}\left(k\right)\right)}{\max\left(x_{i}\left(k\right)\right)-\;\min\left(x_{i}\left(k\right)\right)} \end{array}$$

where *i* = 1,…, *m*; *k* = 1,…, *n*, m is the number of experimental data and *n* is the number of responses. *x*_*i*_ (*k*) denotes the original value of *k*^*th*^ response for *i*^*th*^ experimental run, *x*_*i*_^∗^ (*k*) denotes the normalized value after the data preprocessing, max (*x*_*i*_(*k*)) denotes the largest value of *x*_*i*_(*k*), and min (*x*_*i*_(*k*)) denotes the smallest value of *x*_*i*_(*k*). The next step is to calculate the grey relational coefficient, *ξ*_*i*_(*k*), from the normalized values by using the following equation (Table 5):

(10) $$\begin{array}{c} \xi_{i}\left(k\right)=\frac{\Delta_{\min}-\xi \Delta_{\max}}{\Delta_{0i}k-\xi \Delta_{\max}} \end{array}$$

where Δ_0*i*_ is the deviation value obtained from the reference value (*x*_0_(*k*)) and the comparability value (*x*_*i*_(*k*)).

(11) $$\begin{array}{c} \Delta_{0i}=\left\|x_{0}\left(k\right)-x_{i}\left(k\right)\right\| \end{array}$$

Δ_min_ and Δ_max_ are the minimum and maximum values of the absolute difference (Δ_0*i*_). *ξ* is the distinguishing coefficient, where *ξ*∈[0, 1] and value 0.5 is used for experimentation [7]. The next step is to find out the grey relational grade (GRG) using the following equation (Table 5):

(12) $$\begin{array}{c} \gamma_{i}=\frac{1}{n}\overset{n}{\underset{k=1}{\sum }}\xi_{i}\left(k\right) \end{array}$$

where *γ*_*i*_ is the required grey relational grade for the *i*^*th*^ experiment. The results are utilized for optimizing the multiresponses as they are converted into a single grade.

**Figure 3:**
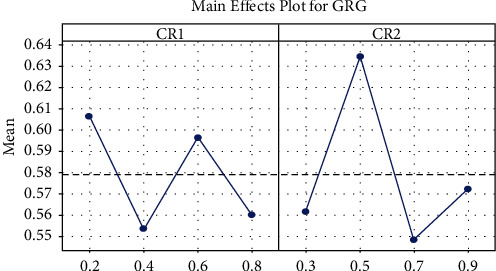
Main effect plot of grey relational grade.

From the value of GRG, the effects of each process parameter at different levels are plotted and shown in Figure 3. Using these results optimal settings for the parameters *CR*_1_ and *CR*_2_ are derived as 0.2 and 0.5, respectively.

### 4.3. Analysis of the Results

The proposed algorithm (MoD-ProM) is run for the real life and for the synthetic datasets and the values for quality dimensions, namely, completeness (*f*_*c*_), preciseness (*f*_*p*_), simplicity (*f*_*s*_), and generalization (*f*_*g*_), for the discovered nondominated solutions are shown in Tables 6 and 7, respectively.

The proposed approach is compared with the NSGA-II algorithm for process discovery. Tables 7 and 8 present the values for the quality dimensions for the discovered nondominated solutions for real-life and synthetic datasets, respectively.

**Figure 4:**
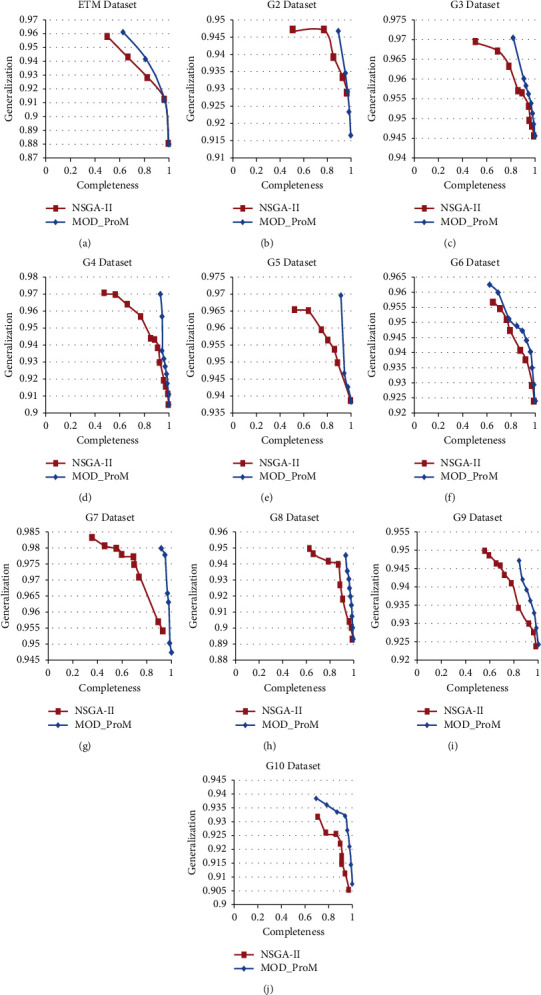
Pareto-curve for the nondominated solutions for NSGA-II and MoD-ProM for synthetic datasets, namely (a) ETM dataset (b) G2 dataset (c) G3 dataset (d) G4 dataset (e) G5 dataset (f) G6 dataset (g) G7 dataset (h) G8 dataset (i) G9 dataset (j) G10 dataset.

**Figure 5:**
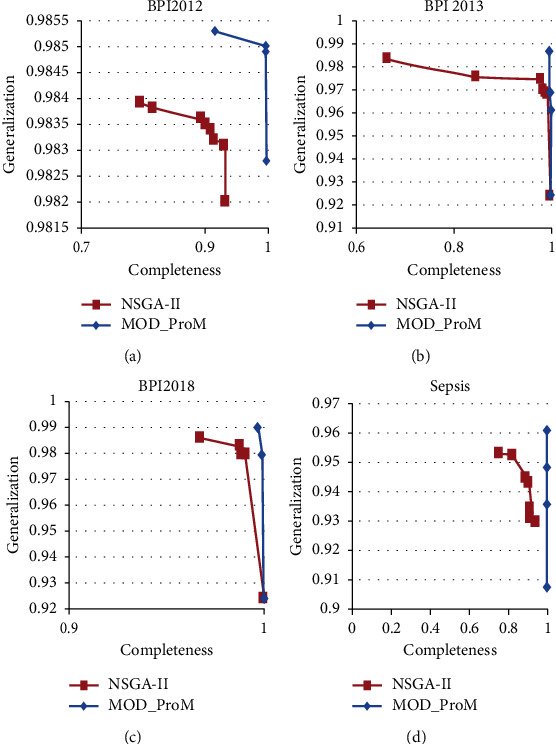
Pareto-curve for the nondominated solutions for NSGA-II and MoD-ProM for real-life datasets, namely (a) BPI 2012 dataset (b) BPI 2013 dataset (c) BPI 2018 dataset (d) Sepsis dataset.

Pareto-curves for the nondominated solutions of NSGA-II and the proposed multiobjective differential evolution for process mining (MoD-ProM) are plotted for comparison (Figures 4 and 5). The Pareto-curves show that in 12 out of 14 datasets, the results of the proposed algorithm are superior to the NSGA-II algorithm.

**Figure 6:**
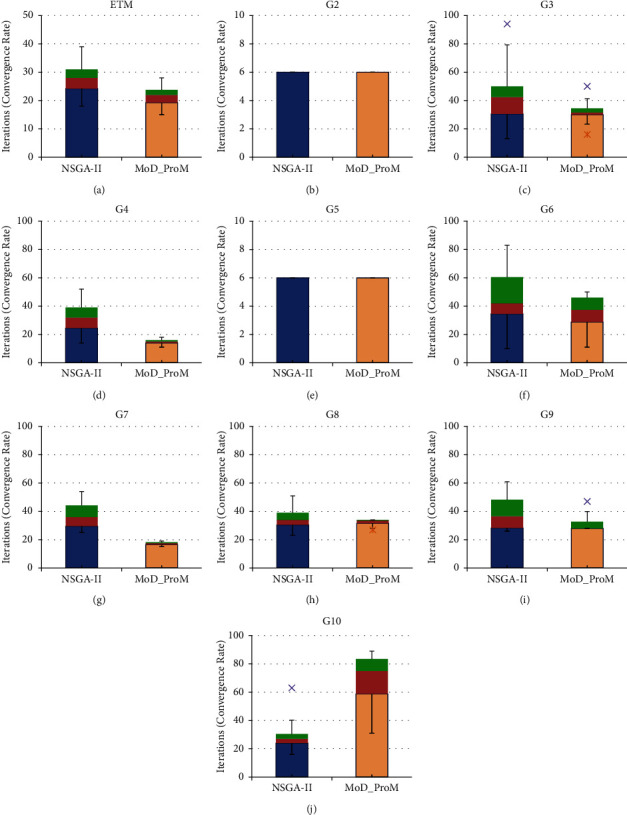
Convergence rate for NSGA-II and MoD-ProM for synthetic datasets, namely (a) ETM dataset (b) G2 dataset (c) G3 dataset (d) G4 dataset (e) G5 dataset (f) G6 dataset (g) G7 dataset (h) G8 dataset (i) G9 dataset (j) G10 dataset.

**Figure 7:**
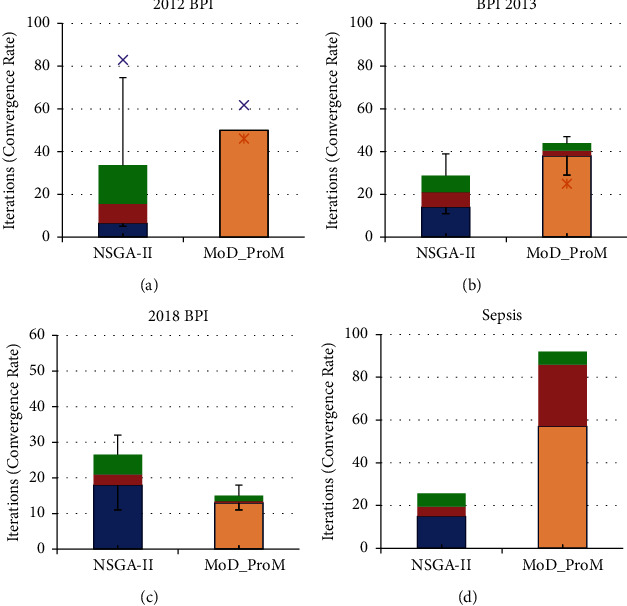
Convergence rate for NSGA-II and MoD-ProM for real-life datasets, namely (a) BPI 2012 dataset (b) BPI 2013 dataset (c) BPI 2018 dataset (d) Sepsis dataset.

**Figure 8:**
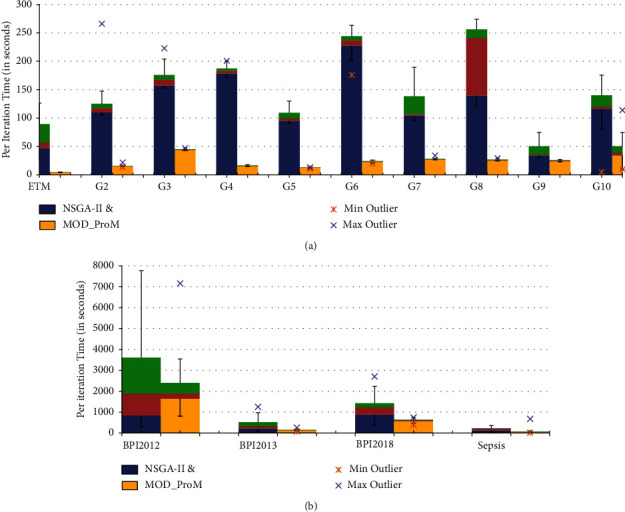
Comparison of per iteration running time for NSGA-II/MoD-ProM, over 30 runs. (a) Synthetic dataset. (b) Real-life dataset.

We also compute the convergence rate and per iteration computation time for NSGA-II and the proposed MoD-ProM, over 30 runs (Figures 6, 7, 8). While in 2 datasets, the algorithms (NSGA-II, MoD-ProM) show a similar convergence rate, and in 8 out of 14 datasets, the proposed MoD-ProM converges faster than NSGA-II, demonstrating superior exploration of the proposed approach.  Figure 8 shows that in all cases, the proposed algorithm is superior to NSGA-II in terms of running time per iteration. It is evident from the results that NSGA-II is computationally more expensive than the proposed MoD-ProM algorithm.

The proposed algorithm is also compared with Genetic Miner, Heuristic Miner, *α*^++^, ILP, and Inductive Miner. To rank the proposed approach and the traditional algorithms, additional comparison based on a weighted average [22] of the quality dimensions is made (Table 9). Reference [22] proposed a weighted average computation methodology suitable to the process mining domain as follows:

(13) $$\begin{array}{c} \text{Weighted Sum}=\frac{\left(10*f_{c}+\left(1*f_{p}\right)+\left(1*f_{s}\right)+\left(1*f_{g}\right)\right)}{13} \end{array}$$

where for a given process model, *f*_*c*_, *f*_*p*_, *f*_*s*_, and *f*_*g*_ denote the completeness, preciseness, simplicity, and generalization values, respectively. A higher weight is assigned to completeness as the process model should be able to reproduce the behavior expressed in the event log.

Table 10 shows the quality dimensions for the process model discovered by the state-of-the-art algorithms. The results (Table 9) show that the proposed algorithm produces superior-quality process models for all the datasets in terms of the weighted average.

It is also observed that the models generated through the optimization of a combination of completeness and generalization exhibit superior values for the other quality dimensions.

#### 4.3.1. Process Model Representation

As discussed earlier (Section on Initialization), the proposed approach represents a process model as a causality relation matrix [39]. However, many state-of-the-art approaches use other semantics, such as Petri net, BPMN models, and DFGs. Petri net is possibly the more popular technique for visualizing the discovered process model. We apply the methodology given by [39] to map between a Petri net and a causality relation matrix.

**Figure 9:**
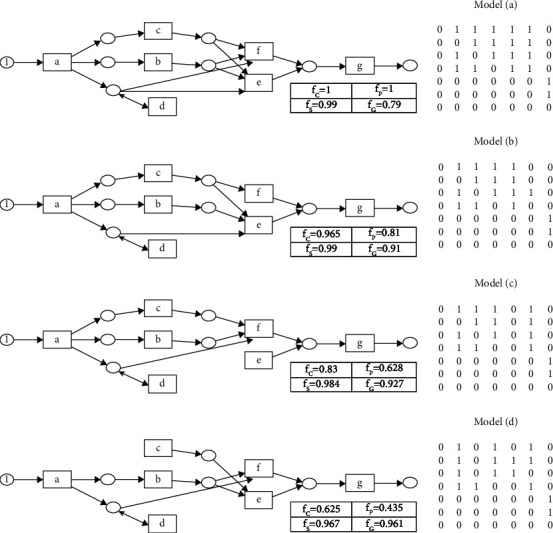
Petri net and causality relation matrix of nondominated process models of the proposed algorithm for the ETM dataset.

To better explain our results, we have graphically depicted the discovered models (causality relation matrices) as Petri nets for the ETM event log. ETM is a popular dataset in the literature comprising seven tasks. Being a small dataset, it is feasible to show (Figure 9) the causality relation matrices and the corresponding Petri nets of the four models discovered by the proposed algorithm (MoD-ProM).

**Figure 10:**
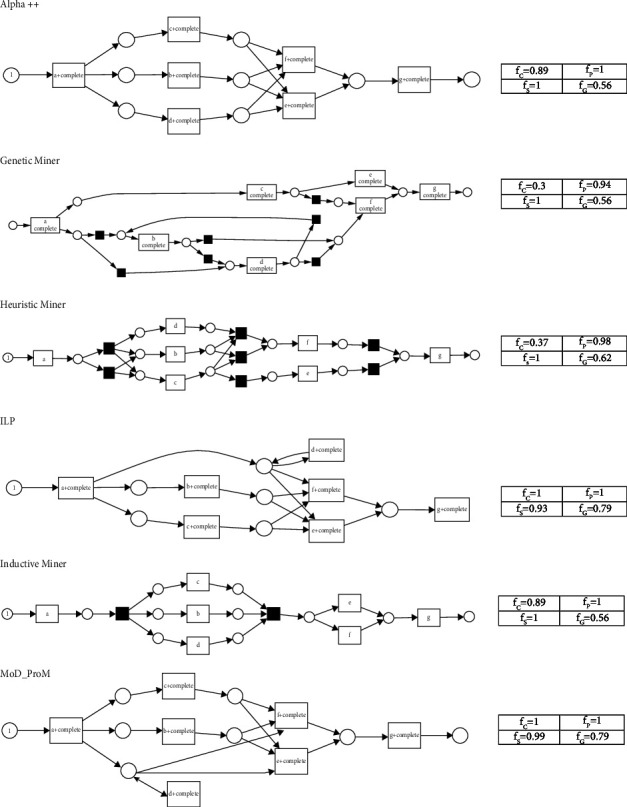
Petri net of the process models discovered by the compared algorithms for the ETM dataset.

For the ETM dataset, the ProM tool generated Petri nets for the state-of-the-art algorithms is shown in Figure 10. To compare with the proposed approach, the Petri net of the model with the highest completeness value, discovered by the proposed MoD-ProM, is also drawn in Figure 10.

The completeness or replay fitness [3] quantifies the ability to replay the trace from an event log onto the Petri net [15]. That is, a process model (Petri net) will exhibit a perfect completeness value if every process instance in the given event log can be replayed (simulated) in the Petri net. For the ETM dataset (Figure 10), it is observed that the proposed MoD-ProM algorithm and the ILP algorithm can replay every process instance in the event log. It is observed that the process model discovered by inductive miner and *α*^++^ does not replay some of the traces, such as (a, b, c, f, g) and (a, c, d, f, g). Traces (a, b, c, d, e, g) and (a, c, b, d, f, g) are not replayed by the model generated by the heuristic miner algorithm. Similarly, the process model generated by the genetic miner algorithm does not replay (a, c, d, b, f, g) and (a, d, c, b, f, g).

## 5. Conclusions and Future Works

While conventional process mining algorithms generate a unique process model from the event logs, multiobjective evolutionary algorithms generate several candidate models. The goodness of the generated process models is measured based on quality dimensions such as completeness, generalization, simplicity, and preciseness. A practitioner in the field of process mining may select the most appropriate process model based on the domain requirement. For example, if a user requires a model that replays the maximum number of traces, he/she may pick the model with a better value of completeness [5].

In this paper, we are using the idea of differential evolution towards generating a Pareto-front in the domain of process discovery, a first attempt in this direction. The proposed algorithm performs optimization using completeness and generalization as objective functions. These two quality dimensions make a good pair, as a model with high generalization value can help in improving the current system and can be used for designing future improved processes. Completeness is an important quality dimension because a discovered process model is expected to describe the behavior stored in the log.

The experiments were run for ten synthetic and four real-life datasets and are repeated 30 times for each dataset. The results are compared with state-of-the-art process discovery algorithms such as *α*^++^, heuristic miner, genetic miner, ILP, and inductive miner, and also with NSGA-II for process discovery.

Results show that the models generated by the proposed approach vis-a-vis the compared approaches exhibit a higher value for all the quality dimensions indicating the discovery of “good” process models. The nondominated solutions generated by the proposed approach (MoD-ProM) are better than those generated by the NSGA-II algorithm for process discovery. The Pareto-curve shows that the results of the proposed algorithm are superior or at least as good as those of the NSGA-II algorithm. In terms of computational time requirement, the MoD-ProM algorithm performs consistently better for all datasets as compared to the NSGA-II algorithm.

In summary, we present a novel proposal for process model discovery. The approach employs a multiobjective differential evolution method to optimize the novel combination of completeness and generalization. Results show that the proposed approach is computationally efficient in discovering good-quality process models. However, the proposed approach is limited by the hardware availability.

In the future, we plan to evaluate the applicability of recent multiobjective algorithms [51, 52] in the domain of process discovery and study their computational complexity. In addition, to address the computational intensity and time consumption of process discovery for large event logs, we can explore parallel implementations (multicore processors, GPU-based processing, and distributed computing environments) for the proposed algorithm.

## Figures and Tables

**Algorithm 1:**
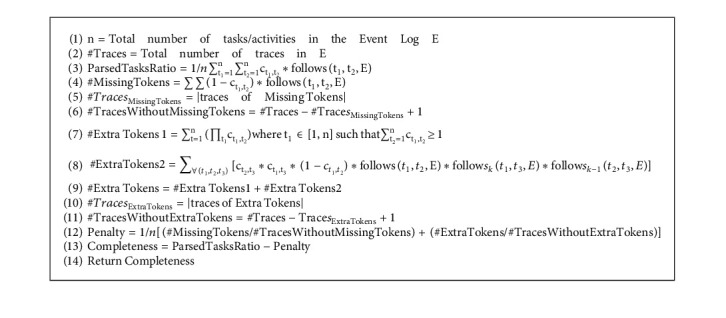
Completeness (C, E).

**Algorithm 2:**
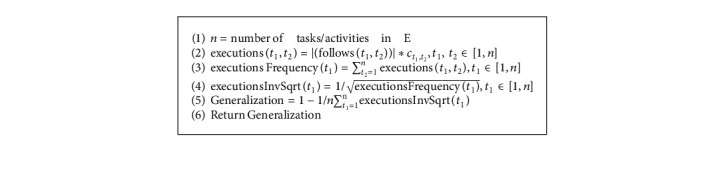
Generalization computation: Generalization (C, E).

**Algorithm 3:**
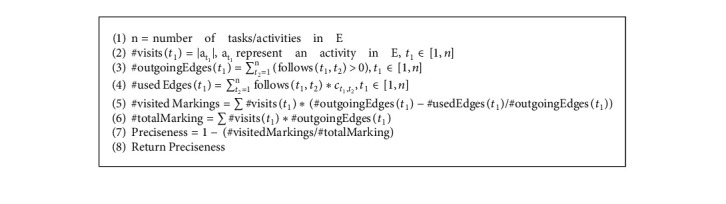
Preciseness (C, E).

**Algorithm 4:**
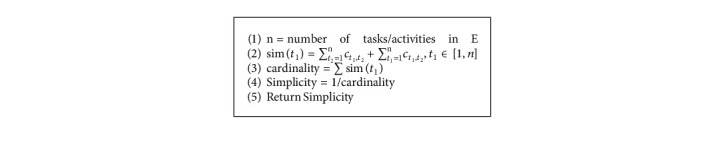
Simplicity (C, E).

**Algorithm 5:**
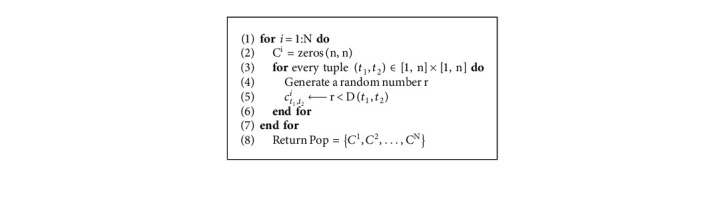
Computation of the initial population of N individuals, each of size *n* × *n*: Initialize Population (*n*, *N*, *D*).

**Algorithm 6:**
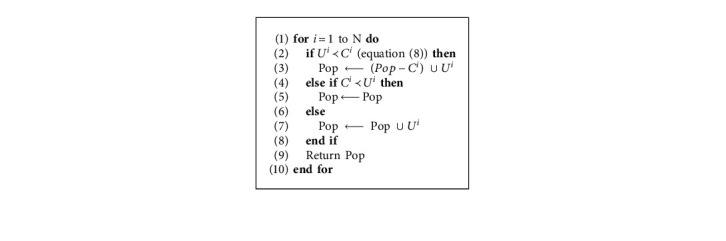
Selection of the fittest individuals from current (Pop) and candidate (*Pop*_*U*_) populations, each of size N: Selection (*Pop*_*U*_, Pop).

**Algorithm 7:**
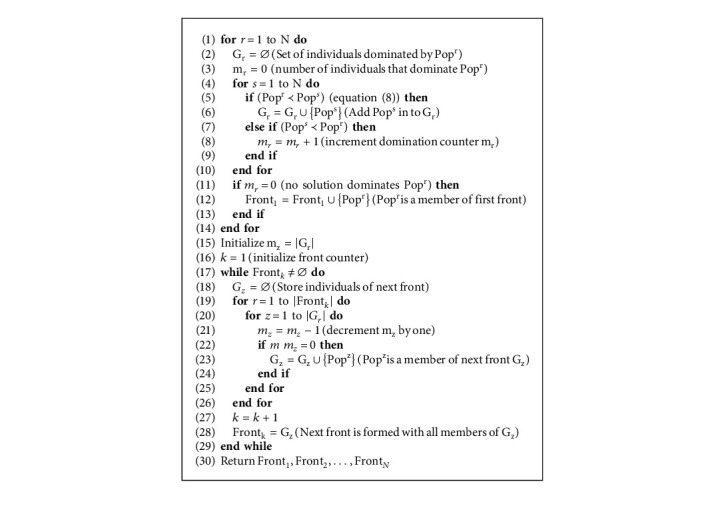
Non-dominated sorting algorithm: Nondominated Sorting (Pop, N).

**Algorithm 8:**
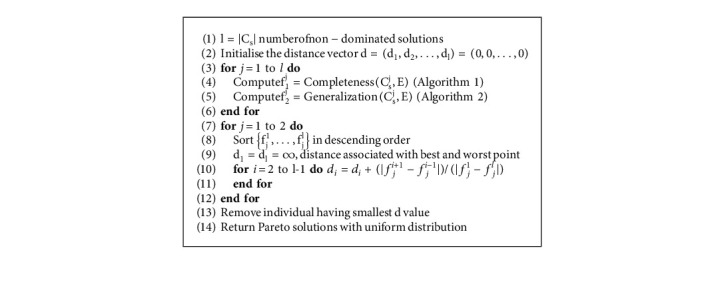
Pseudo-code for crowding distance algorithm: Crowding distance (*C*_*s*_, *E*), *C*_*s*_: Nondominated solutions.

**Algorithm 9:**
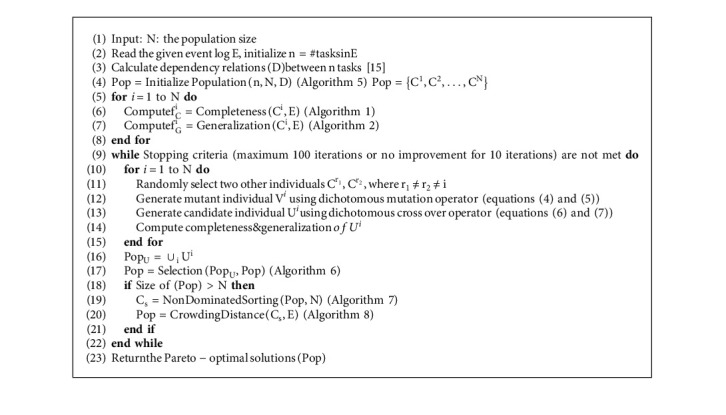
Pseudo code for multiobjective differential approach for process mining (MoD-ProM).

**Table 1:** An event log with three process instances.

Case ID	Process instance
101	*T*_1_ *T*_2_ *T*_3_
102	*T*_1_ *T*_2_ *T*_4_ *T*_6_ *T*_5_ *T*_7_
103	*T*_1_ *T*_2_ *T*_4_ *T*_5_ *T*_6_ *T*_7_

**Table 2:** Details of datasets used in experiments.

Type	Event log	Tasks	Traces	Events	Source
Synthetic event logs	ETM	7	100	790	[15, 44]
	g2	22	300	4501	
	g3	29	300	14599	
	g4	29	300	5975	
	g5	20	300	6172	
	g6	23	300	5419	
	g7	29	300	14451	
	g8	30	300	5133	
	g9	26	300	5679	
	g10	23	300	4117	

Real-life event logs	BPI 2012	23	13087	262200	[45]
	BPI 2013-incident	13	7554	65533	[46]
	BPI 2018-reference	6	43802	128554	[47]
	Sepsis	16	1050	150000	[48]

**Table 3:** Experimental results for completeness and generalization as per Taguchi L16 Orthogonal Array.

Run No.	Process parameters		Experimental results	
	CR1	CR2	Completeness	Generalization
1	0.2	0.3	0.9959	0.8931
2	0.2	0.5	0.9996	0.8927
3	0.2	0.7	0.99531	0.8926
4	0.2	0.9	0.9943	0.8919
5	0.4	0.3	0.9945	0.8917
6	0.4	0.5	0.998	0.8928
7	0.4	0.7	0.9971	0.8922
8	0.4	0.9	0.9973	0.8929
9	0.6	0.3	0.9958	0.892
10	0.6	0.5	0.9961	0.893
11	0.6	0.7	0.9994	0.8926
12	0.6	0.9	0.9965	0.8925
13	0.8	0.3	0.9984	0.8929
14	0.8	0.5	0.995	0.8915
15	0.8	0.7	0.994	0.8923
16	0.8	0.9	0.9993	0.8924

**Table 4:** Grey relational generation values.

Run No.	Completeness	Generalization
1	0.339	1.000
2	1.000	0.750
3	0.234	0.687
4	0.054	0.250
5	0.089	0.125
6	0.714	0.813
7	0.554	0.438
8	0.589	0.875
9	0.321	0.313
10	0.375	0.938
11	0.964	0.687
12	0.446	0.625
13	0.786	0.875
14	0.179	0.000
15	0.000	0.500
16	0.946	0.562

**Table 5:** Grey relational coefficient and grey relational grade values.

Run No	Evaluation of Δ_0*i*_		Grey relational coefficient		GRG	Rank
	Completeness	Generalization	Completeness	Generalization		
Ideal sequence	1	1	1	1		
1	0.661	0.000	0.431	1.000	0.715	5
2	0.000	0.250	1.000	0.667	0.833	1
3	0.766	0.313	0.395	0.615	0.505	10
4	0.946	0.750	0.346	0.400	0.373	14
5	0.911	0.875	0.354	0.364	0.359	15
6	0.286	0.187	0.636	0.727	0.682	6
7	0.446	0.562	0.528	0.471	0.499	11
8	0.411	0.125	0.549	0.800	0.675	7
9	0.679	0.687	0.424	0.421	0.423	12
10	0.625	0.062	0.444	0.889	0.667	8
11	0.036	0.313	0.933	0.615	0.774	2
12	0.554	0.375	0.475	0.571	0.523	9
13	0.214	0.125	0.700	0.800	0.750	3
14	0.821	1.000	0.378	0.333	0.356	16
15	1.000	0.500	0.333	0.500	0.417	13
16	0.054	0.438	0.903	0.533	0.718	4

**Table 6:** Quality dimensions of nondominated solutions obtained from the MoD-ProM algorithm for synthetic datasets.

ETM				g2				g3				g4				g5			
*f*_*C*_	*f*_*G*_	*f*_*S*_	*f*_*P*_	*f*_*C*_	*f*_*G*_	*f*_*S*_	*f*_*P*_	*f*_*C*_	*f*_*G*_	*f*_*S*_	*f*_*P*_	*f*_*C*_	*f*_*G*_	*f*_*S*_	*f*_*P*_	*f*_*C*_	*f*_*G*_	*f*_*S*_	*f*_*P*_
0.625	0.961	0.967	0.435	0.896	0.946	0.996	0.824	0.818	0.97	1	0.85	0.927	0.969	0.998	0.867	0.916	0.9695	1	0.873
0.813	0.9419	0.984	0.621	0.954	0.934	0.998	0.915	0.906	0.96	1	0.854	0.947	0.957	0.998	0.916	0.946	0.946	1	0.916
0.965	0.911	0.993	0.814	0.972	0.929	0.998	0.941	0.927	0.958	1	0.872	0.948	0.936	0.999	0.944	0.974	0.942	1	0.953
1	0.79	0.994	1	0.988	0.923	0.999	0.979	0.947	0.956	1	0.893	0.96	0.932	1	0.953	1	0.938	1	1
				1	0.916	1	1	0.966	0.953	1	0.915	0.971	0.927	1	0.96				
								0.978	0.951	1	0.956	0.982	0.923	1	0.972				
								0.99	0.948	1	0.978	0.988	0.917	1	0.984				
								1	0.945	1	1	0.994	0.911	1	0.992				
												1	0.904	1	1				

g6				g7				g8				g9				g10			
*f*_*C*_	*f*_*G*_	*f*_*S*_	*f*_*P*_	*f*_*C*_	*f*_*G*_	*f*_*S*_	*f*_*P*_	*f*_*C*_	*f*_*G*_	*f*_*S*_	*f*_*P*_	*f*_*C*_	*f*_*G*_	*f*_*S*_	*f*_*P*_	*f*_*C*_	*f*_*G*_	*f*_*S*_	*f*_*P*_

0.621	0.9623	1	0.685	0.92	0.979	1	0.902	0.933	0.945	1	0.893	0.842	0.947	1	0.754	0.697	0.938	1	0.767
0.691	0.959	1	0.667	0.947	0.977	1	0.945	0.948	0.935	1	0.932	0.871	0.942	1	0.795	0.787	0.936	1	0.835
0.781	0.951	1	0.743	0.967	0.965	1	0.975	0.96	0.93	1	0.943	0.907	0.939	1	0.83	0.872	0.933	1	0.86
0.844	0.948	1	0.772	0.979	0.963	1	0.981	0.968	0.925	1	0.963	0.937	0.936	1	0.83	0.937	0.932	1	0.799
0.895	0.947	1	0.766	0.989	0.95	1	0.994	0.976	0.919	1	0.983	0.965	0.933	1	0.829	0.959	0.927	1	0.875
0.928	0.943	1	0.798	1	0.947	1	1	0.984	0.914	1	0.987	0.984	0.928	1	0.945	0.976	0.921	1	0.935
0.96	0.94	1	0.906					0.989	0.907	1	0.989	1	0.924	1	1	0.988	0.914	1	0.967
0.973	0.934	1	0.945					0.995	0.9	1	0.993					1	0.907	1	1
0.986	0.929	1	0.993					0.999	0.893	1	1								
1	0.923	1	1																

*f*_*C*_: Completeness, *f*_*P*_: Preciseness, *f*_*S*_: Simplicity, *f*_*G*_: Generalization.

**Table 7:** Quality dimensions of nondominated solutions obtained by NSGA-II for process mining and MoD-ProM algorithm for real-life datasets.

Nondominated solutions obtained from NSGA-II for process mining algorithm															
BPI 2012				BPI 2013				BPI 2018				Sepsis			
*f*_*C*_	*f*_*G*_	*f*_*S*_	*f*_*P*_	*f*_*C*_	*f*_*G*_	*f*_*S*_	*f*_*P*_	*f*_*C*_	*f*_*G*_	*f*_*S*_	*f*_*P*_	*f*_*C*_	*f*_*G*_	*f*_*S*_	*f*_*P*_
0.794	0.9839	0.9984	0.6945	0.6637	0.9834	0.9964	0.6956	0.9671	0.9857	0.9461	0.406	0.7509	0.9529	0.9955	0.499
0.815	0.9838	0.9986	0.6756	0.8453	0.9758	0.9953	0.6433	0.9873	0.9824	0.9568	0.5989	0.8198	0.9524	0.9958	0.538
0.892	0.9836	0.9987	0.6819	0.9749	0.975	0.997	0.7879	0.9886	0.9792	0.9848	0.7978	0.8872	0.9448	0.9979	0.664
0.899	0.9835	0.9989	0.7151	0.9816	0.9749	0.9971	0.805	0.99	0.979	0.9768	0.7922	0.9033	0.943	0.9974	0.649
0.907	0.9834	0.999	0.6688	0.9832	0.9699	0.9973	0.8308	0.9998	0.9236	0.9907	0.9855	0.9158	0.9345	0.9981	0.704
0.914	0.9832	0.9991	0.6874	0.9871	0.9685	0.9985	0.8818					0.9165	0.931	0.9977	0.670
0.929	0.9831	0.9992	0.7271	0.9916	0.9683	0.9988	0.8915					0.9434	0.9299	0.998	0.714
0.931	0.982	0.9993	0.7056	0.9966	0.9241	0.9983	0.869								
				0.9974	0.9239	0.9987	0.8458								

Non-dominated solutions obtained from MoD-ProM algorithm															
BPI 2012				BPI 2013				BPI 2018				Sepsis			

*f*_*C*_	*f*_*G*_	*f*_*S*_	*f*_*P*_	*f*_*C*_	*f*_*G*_	*f*_*S*_	*f*_*P*_	*f*_*C*_	*f*_*G*_	*f*_*S*_	*f*_*P*_	*f*_*C*_	*f*_*G*_	*f*_*S*_	*f*_*P*_
0.9146	0.9853	0.9995	0.9229	0.9942	0.9768	0.998	0.7398	0.9965	0.9793	0.985	0.806	0.994	0.961	0.9994	0.849
0.9957	0.985	0.9996	0.9294	0.9946	0.97	0.9979	0.8078	0.9986	0.9792	0.9848	0.7978	0.9957	0.949	0.9995	0.892
0.9965	0.9849	0.9997	0.930	0.9948	0.9696	0.9985	0.8312	0.999	0.979	0.981	0.792	0.9973	0.936	0.9997	0.941
0.9974	0.9828	0.9998	0.9310	0.996	0.9687	0.9989	0.8548	0.9998	0.9236	0.993	0.9858	0.9977	0.908	0.9998	0.972
				0.9961	0.9686	0.999	0.8543								
				0.9981	0.9618	0.9996	0.9524								
				0.9987	0.9617	0.9994	0.8672								
				0.9988	0.9245	0.9995	0.8791								

*f*_*C*_: Completeness, *f*_*P*_: Preciseness, *f*_*S*_: Simplicity, *f*_*G*_: Generalization.

**Table 8:** Quality dimensions of nondominated solutions obtained by NSGA-II in process mining domain for synthetic datasets.

ETM				g2				g3				g4				g5			
*f*_*C*_	*f*_*G*_	*f*_*S*_	*f*_*P*_	*f*_*C*_	*f*_*G*_	*f*_*S*_	*f*_*P*_	*f*_*C*_	*f*_*G*_	*f*_*S*_	*f*_*P*_	*f*_*C*_	*f*_*G*_	*f*_*S*_	*f*_*P*_	*f*_*C*_	*f*_*G*_	*f*_*S*_	*f*_*P*_
0.505	0.957	0.948	0.371	0.515	0.947	0.987	0.497	0.514	0.969	0.996	0.515	0.48	0.97	0.984	0.372	0.529	0.965	0.986	0.525
0.67	0.942	0.959	0.371	0.775	0.947	0.991	0.671	0.698	0.966	1	0.663	0.532	0.97	0.978	0.396	0.643	0.964	0.992	0.673
0.83	0.927	0.984	0.628	0.858	0.94	0.994	0.786	0.791	0.963	0.998	0.716	0.568	0.969	0.982	0.412	0.755	0.959	0.996	0.753
0.965	0.911	0.993	0.814	0.936	0.933	0.997	0.888	0.863	0.956	1	0.799	0.666	0.963	0.985	0.532	0.809	0.956	0.822	0.796
1	0.79	0.994	1	0.97	0.928	0.998	0.953	0.899	0.956	1	0.845	0.774	0.956	0.992	0.674	0.862	0.953	0.999	0.836
				1	0.916	1	1	0.95	0.952	1	0.862	0.856	0.943	0.994	0.733	0.892	0.949	1	0.879
								0.956	0.949	1	0.87	0.893	0.943	0.996	0.784	1	0.938	1	1
								0.98	0.947	1	0.91	0.915	0.937	0.997	0.857				
								0.995	0.945	1	0.966	0.931	0.929	0.998	0.926				

g6				g7				g8				g9				g10			
*f*_*C*_	*f*_*G*_	*f*_*S*_	*f*_*P*_	*f*_*C*_	*f*_*G*_	*f*_*S*_	*f*_*P*_	*f*_*C*_	*f*_*G*_	*f*_*S*_	*f*_*P*_	*f*_*C*_	*f*_*G*_	*f*_*S*_	*f*_*P*_	*f*_*C*_	*f*_*G*_	*f*_*S*_	*f*_*P*_

0.654	0.961	0.997	0.504	0.36	0.982	0.989	0.433	0.634	0.949	0.993	0.59	0.565	0.949	1	0.548	0.714	0.931	1	0.583
0.712	0.954	1	0.576	0.464	0.98	0.989	0.468	0.666	0.945	0.994	0.604	0.599	0.948	1	0.641	0.781	0.925	1	0.535
0.765	0.95	1	0.647	0.559	0.979	0.994	0.577	0.792	0.941	0.996	0.677	0.661	0.946	1	0.555	0.868	0.925	1	0.647
0.799	0.946	1	0.72	0.601	0.977	0.992	0.588	0.876	0.939	0.997	0.759	0.693	0.945	1	0.561	0.898	0.922	1	0.647
0.884	0.94	1	0.721	0.698	0.977	0.996	0.678	0.89	0.926	0.998	0.802	0.726	0.943	1	0.643	0.916	0.917	1	0.676
0.926	0.937	1	0.751	0.704	0.974	0.996	0.642	0.912	0.918	0.999	0.819	0.787	0.94	1	0.61	0.917	0.914	1	0.739
0.978	0.928	1	0.831	0.745	0.97	0.996	0.672	0.965	0.903	1	0.92	0.846	0.934	1	0.717	0.942	0.911	1	0.667
0.994	0.923	1	0.871	0.896	0.958	1	0.854	0.989	0.899	1	0.945	0.924	0.929	1	0.754	0.971	0.905	1	0.788
				0.937	0.954	1	0.872	0.995	0.892	1	0.945	0.966	0.927	1	0.787	0.976	0.905	1	0.805
												0.984	0.923	1	0.89				

*f*_*C*_: Completeness, *f*_*P*_: Preciseness, *f*_*S*_: Simplicity, *f*_*G*_: Generalization.

**Table 9:** Weighted sum (equation (13)) of the quality dimensions [22] and the resulting algorithm ranks for each dataset. Rank 7 indicates the best solution.

Event log	Weighted sum							Rank						
	GM	HM	*α* ^++^	ILP	Inductive miner	NSGA-II	MoD-ProM	GM	HM	*α* ^++^	ILP	Inductive miner	NSGA-II	MoD-ProM
ETM	0.423	0.485	0.881	0.978	0.881	0.983	0.983	1	2	3.5	5	3.5	6.5	6.5
g2	0.993	0.993	0.435	0.991	0.94	0.994	0.994	4.5	4.5	1	3	2	6.5	6.5
g3	0.429	0.991	0.132	0.986	0.77	0.989	0.996	2	6	1	4	3	5	7
g4	0.675	0.816	0.991	0.99	0.71	0.993	0.993	1	3	5	4	2	6.5	6.5
g5	0.994	0.994	0.994	0.994	0.81	0.995	0.995	3.5	3.5	3.5	3.5	1	6.5	6.5
g6	0.984	0.722	0.546	0.963	0.64	0.98	0.994	6	3	1	4	2	5	7
g7	0.993	0.984	0.143	0.988	0.756	0.99	0.996	6	3	1	4	2	5	7
g8	0.334	0.618	0.473	0.966	0.795	0.984	0.992	1	3	2	5	4	6	7
g9	0.578	0.776	0.552	0.972	0.679	0.974	0.994	2	4	1	5	3	6	7
g10	0.561	0.8	0.602	0.941	0.6	0.959	0.993	1	4	3	5	2	6	7

**Table 10:** Quality dimensions of the models using the state-of-the-art algorithms.

		ETM	g2	g3	g4	g5	g6	g7	g8	g9	g10
Genetic miner	*f*_*C*_	0.3	1	0.31	0.59	1	1	1	0.26	0.48	0.48
	*f*_*P*_	0.94	1	0.6	1	1	1	1	0.15	1	1
	*f*_*S*_	1	1	1	0.97	1	1	1	0.72	0.96	0.88
	*f*_*G*_	0.56	0.91	0.88	0.90	0.921	0.80	0.91	0.88	0.75	0.61

Heuristic miner	*f*_*C*_	0.37	1	1	0.78	1	0.66	1	0.52	0.74	0.78
	*f*_*P*_	0.98	1	1	1	1	0.99	1	1	1	1
	*f*_*S*_	1	1	1	1	1	0.99	0.98	0.93	0.96	1
	*f*_*G*_	0.62	0.913	0.89	0.81	0.92	0.80	0.81	0.90	0.73	0.60

*α* ^++^	*f*_*C*_	0.89	0.33	0	1	1	0.45	0	0.35	0.48	0.563
	*f*_*P*_	1	0.96	0.18	0.97	1	1	0.12	1	1	1
	*f*_*S*_	1	0.78	0.79	1	1	0.76	0.93	0.74	0.79	0.76
	*f*_*G*_	0.56	0.62	0.74	0.91	0.92	0.84	0.81	0.91	0.59	0.43

ILP	*f*_*C*_	1	1	1	1	1	1	1	1	1	1
	*f*_*P*_	1	0.97	0.97	1	1	0.99	1	0.98	0.98	0.95
	*f*_*S*_	0.93	0.93	0.92	0.96	1	0.74	0.93	0.667	0.9	0.68
	*f*_*G*_	0.79	0.99	0.93	0.91	0.921	0.79	0.91	0.92	0.76	0.61

Inductive miner	*f*_*C*_	0.89	0.958	0.757	0.70	0.80	0.63	0.74	0.79	0.668	0.61
	*f*_*P*_	1	0.89	0.73	0.56	0.75	0.41	0.64	0.637	0.423	0.26
	*f*_*S*_	1	0.9	0.82	0.81	0.9	0.7	0.85	0.63	0.84	0.65
	*f*_*G*_	0.56	0.91	0.94	0.91	0.94	0.91	0.95	0.91	0.88	0.9

*f*_*C*_: Completeness, *f*_*P*_: Preciseness, *f*_*S*_: Simplicity, *f*_*G*_: Generalization.

## Data Availability

Previously reported data (event logs) were used to support this study and are described in the section on Experimentation. These prior studies (and datasets) are cited at relevant places within the text (References [15, 44–47]).

## References

[1] Mamudu A., Bandara W., Wynn M. T., Leemans S. J. J. (2024). Process mining success factors and their interrelationships. *Business & Information Systems Engineering*.

[2] Hejazi S. M., Zandieh M., Mirmozaffari M. (2023). A multi-objective medical process mining model using event log and causal matrix. *Healthcare Analytics*.

[3] Van der Aalst W. M. P. (2016). *Process Mining: Data Science in Action*.

[4] Mining P. (2011). Discovery, conformance and enhancement of business processes. *Springer-Verlag*.

[5] Kumar N., Agarwal M., Deshmukh S., Gupta S. (2020). Moea for discovering pareto-optimal process models: an experimental comparison. *International Journal of Computational Science and Engineering*.

[6] Peng H., Wu Z., Shao P., Deng C. (2016). Dichotomous binary differential evolution for knapsack problems. *Mathematical Problems in Engineering*.

[7] Panda A., Sahoo A., Rout A. K. (2016). Multi-attribute decision making parametric optimization and modeling in hard turning using ceramic insert through grey relational analysis: a case study. *Decision Science Letters*.

[8] Lin C. L. (2004). Use of the taguchi method and grey relational analysis to optimize turning operations with multiple performance characteristics. *Materials and Manufacturing Processes*.

[9] Deb K., Pratap A., Agarwal S., Meyarivan T. A. M. T. (2002). A fast and elitist multiobjective genetic algorithm: nsga-ii. *IEEE Transactions on Evolutionary Computation*.

[10] Van der Aalst W., Weijters T., Maruster L. (2004). Workflow mining: discovering process models from event logs. *IEEE Transactions on Knowledge and Data Engineering*.

[11] Alves de Medeiros A. K., Van Dongen B. F., Van Der Aalst W. M. P., Weijters A. J. M. M. (2004). Process mining: extending the *α*-algorithm to mine short loops. *Technical report*.

[12] Boudewijn F. V. D., Van der Aalst W. M. P. Multi-phase process mining: building instance graphs.

[13] Boudewijn F. V. D., Van der Aalst W. M. P. Multi-phase process mining: aggregating instance graphs into epcs and petri nets.

[14] Weijters A. J. M. M., van Der Aalst W. M. P., Alves De Medeiros A. K. (2006). Process mining with the heuristics miner-algorithm. *Technische Universiteit Eindhoven*.

[15] Ana Karla Alves de Medeiros (2006). *Genetic process mining*.

[16] Wen L., van der Aalst W. M. P., Wang J., Sun J. (2007). Mining process models with non-free-choice constructs. *Data Mining and Knowledge Discovery*.

[17] Wen L., Wang J., Sun J. (2007). Mining invisible tasks from event logs. *Lecture Notes in Computer Science*.

[18] Li J., Liu D., Yang B. Process mining: extending *α*-algorithm to mine duplicate tasks in process logs.

[19] Van der Aalst W. M. P., Gunther C. W. Finding structure in unstructured processes: the case for process mining.

[20] Goedertier S., Martens D., Vanthienen J., Baesens B. (2009). Robust process discovery with artificial negative events. *Journal of Machine Learning Research*.

[21] Deshmukh S., Gupta S., Kumar N. Ga-prom: a genetic algorithm for discovery of complete process models from unbalanced logs.

[22] Buijs J. C. A. M., Van Dongen B. F., van der Aalst W. M. P. (2012). On the role of fitness, precision, generalization and simplicity in process discovery. *Lecture Notes in Computer Science*.

[23] Sander J. J. L., Fahland D., van der Aalst W. M. P. Discovering block-structured process models from event logs-a constructive approach.

[24] De Smedt J., De Weerdt J., Vanthienen J. Multi-paradigm process mining: retrieving better models by combining rules and sequences.

[25] Cheng H.-J., Ou-Yang C., Juan Y.-C. (2015). A hybrid approach to extract business process models with high fitness and precision. *Journal of Industrial and Production Engineering*.

[26] vanden Broucke S. K., De Weerdt J. (2017). Fodina: a robust and flexible heuristic process discovery technique. *Decision Support Systems*.

[27] Joos C. A. M. B., van Dongen B. F., van der Aalst W. M. P. Discovering and navigating a collection of process models using multiple quality dimensions.

[28] Srinivas N., Deb K. (1994). Muiltiobjective optimization using nondominated sorting in genetic algorithms. *Evolutionary Computation*.

[29] Storn R., Price K. (1997). Differential evolution–a simple and efficient heuristic for global optimization over continuous spaces. *Journal of Global Optimization*.

[30] Huang Z., Chen Y. (2013). An improved differential evolution algorithm based on adaptive parameter. *Journal of Control Science and Engineering*.

[31] RobicT F. I. L. I. P. IcB. Demo: differentialevolutionfor multiobjectiveoptimization.

[32] Tušar T., Filipič B. Differential evolution versus genetic algorithms in multiobjective optimization.

[33] Li Y., Sansavini G., Zio E. (2013). Non-dominated sorting binary differential evolution for the multi-objective optimization of cascading failures protection in complex networks. *Reliability Engineering and System Safety*.

[34] Xue B., Fu W., Zhang M. Differential evolution (de) for multi-objective feature selection in classification.

[35] Sikdar U. K., Ekbal A., Saha S. (2015). Mode: multiobjective differential evolution for feature selection and classifier ensemble. *Soft Computing*.

[36] Asilian Bidgoli A., Ebrahimpour-Komleh H., Rahnamayan S. A novel multi-objective binary differential evolution algorithm for multi-label feature selection.

[37] Zhang Y., Gong D.-W., Gao X.-Z., Tian T., Sun X.-Y. (2020). Binary differential evolution with self-learning for multi-objective feature selection. *Information Sciences*.

[38] Gupta S., Deshmukh S., Kumar N. (2024). Mantaray-prom: an efficient process model discovery algorithm. *AI Communications*.

[39] de Medeiros A. K. A., Weijters A. J. M. M., van der Aalst W. M. P. (2007). Genetic process mining: an experimental evaluation. *Data Mining and Knowledge Discovery*.

[40] Buijs J. C. A. M., van Dongen B. F., van der Aalst W. M. P. (2014). Quality dimensions in process discovery: the importance of fitness, precision, generalization and simplicity. *International Journal of Cooperative Information Systems*.

[41] Van Eck M. L. (2013). *Alignment-based Process Model Repair and its Application to the Evolutionary Tree Miner*.

[42] van Dongen B. F., Carmona J., Chatain T. A unified approach for measuring precision and generalization based on anti-alignments.

[43] Mannhardt F., De Leoni M., Reijers H. A., Van Der Aalst W. M. P. Measuring the precision of multi-perspective process models.

[44] Vázquez-Barreiros B., Mucientes M., Lama M. (2015). Prodigen: mining complete, precise and minimal structure process models with a genetic algorithm. *Information Sciences*.

[45] Bf Van Dongen and Bpi Challenge (2012). *Event Log of a Loan Application Process*.

[46] Ward S. (2013). Bpi challenge 2013, incidents.

[47] Boudewijn Van (2018). Bpi challenge 2018. https://data.4tu.nl/articles/dataset/BPI_Challenge_2018/12688355/1.

[48] Mannhardt F. (2016). Sepsis cases–event log. https://data.4tu.nl/articles/dataset/Sepsis_Cases_-_Event_Log/12707639.

[49] Broucke S. K. L. M. V., De Weerdt J., Vanthienen J., Baesens B. A comprehensive benchmarking framework (cobefra) for conformance analysis between procedural process models and event logs in prom.

[50] Freddi A., Salmon M. Introduction to the taguchi method.

[51] Abed-alguni B. H., Al-Jarah S. H. (2024). Ibja: an improved binary djaya algorithm for feature selection. *Journal of Computational Science*.

[52] Alawad N. A., Abed-alguni B. H., Al-Betar M. A., Jaradat A. (2023). Binary improved white shark algorithm for intrusion detection systems. *Neural Computing & Applications*.

[53] Deshmukh S., Gupta S., Kumar N. (2024). Multi-objective binary differential approach with parameter tuning for discovering business process models: mod-prom. https://arxiv.org/abs/2406.17713.

